# Chloroplast capture and range extension after hybridization in taro (*Colocasia esculenta*)

**DOI:** 10.1002/ece3.70082

**Published:** 2024-08-27

**Authors:** P. J. Matthews, M. A. Hossain, D. Sookchaloem, V. D. Nguyen, S. Y. Wong, J. Joling, M. E. Schranz, F. T. Bakker, E. Tabuchi, I. Ahmed, A. Hay

**Affiliations:** ^1^ Department of Cross‐Field Research National Museum of Ethnology Suita Japan; ^2^ Department of Genetics and Plant Breeding Bangladesh Agricultural University Mymensingh Bangladesh; ^3^ Department of Forest Biology Kasetsart University Bangkok Thailand; ^4^ Institute for Ecology and Biological Resources & Graduate University of Science and Technology Hanoi Vietnam; ^5^ Institute of Biodiversity and Environmental Conservation, Universiti Malaysia Sarawak Samarahan Sarawak Malaysia; ^6^ Biosystematics Group Wageningen University Wageningen The Netherlands; ^7^ Alpha Genomics Private Limited Islamabad Pakistan; ^8^ Microbiological Analysis Team, Group for Biometrology Korea Research Institute of Standards and Science (KRISS) Daejeon Republic of Korea; ^9^ Jardín Botánico de la Paz y Flora Bitaco Valle del Cauca Colombia

**Keywords:** aroids, crop wild relatives, domestication, evolution, hybrids, introgression

## Abstract

Complete chloroplast genomes of 17 samples from six species of *Colocasia* (Araceae) were sequenced, assembled, and aligned together with two previously reported complete genome sequences from taro (*Colocasia esculenta*). Analysis provides a well‐supported phylogenetic tree for taro and closely‐related wild *Colocasia* species in Southeast Asia. Two chloroplast lineages (CI and CII) form a well‐defined haplotype group and are found in cultivated taros known as var. esculenta (dasheen, CI), var. *antiquorum* (eddoe, CII), and in a widespread, commensal wild form known as var. *aquatilis* (CI). A third lineage (CIII) is also found in wild taros known as var. *aquatilis* and in the wild species *C. lihengiae*, *C. formosana*, and *C. spongifolia*. We suggest three different scenarios to explain the grouping of CIII wild taros (*C. esculenta*) with other wild *Colocasia* species. Chloroplast lineages CI and CIII in *C. esculenta* and an unknown parent species may be involved in an as yet undated history of hybridization, chloroplast capture, and range extension. Substantial taxonomic revision may be needed for *C. esculenta* after further studies of morphological and genetic diversity within the crop, in wild populations, and in closely related wild species. The results also point to the Bengal delta as a region of key interest for future research on the origins of tropical wetland taros.

## Graphical abstract

Complete chloroplast genome sequences from wild and cultivated taro (*Colocasia esculenta*) and closely related *Colocasia* species provide a well‐supported phylogenetic tree with three main chloroplast lineages. Two of these (CI and CIII) appear to be involved in an ancient history of hybridization, chloroplast capture, and range extension. Tropical wetland cultivars (CI, Type I haplotype) may originate in the Bengal delta region, where wild CI sister lineages were found. *C. oresbia*, a geographical isolate in island Southeast Asia (see figure), appears to be a near outgroup.

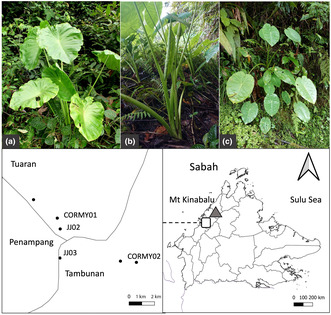

## 1 INTRODUCTION

*Colocasia esculenta* (L.) (Schott, 1858) (taro, Araceae) is an ancient starchy staple and green vegetable crop cultivated in tropical to temperate regions of the world and is consumed today by billions of people (Matthews & Ghanem, 2021). Remains or probable remains of taro (including starch grains, calcium oxalate raphides, pollen, seeds, dried macro‐remains, and volcanic ash impressions) have been reported in archaeological contexts ranging in age from hundreds to thousands of years ago in Asia (GARF, 2003; Li et al., 2022; Paz, 2005), the Pacific (Golson et al., 2017; Horrocks & Thomas, 2022; Loy et al., 1992; Prebble et al., 2019), and Egypt (van der Veen, 2011). Ahmed et al. (2020) argued that if taro was introduced to Papua New Guinea as a cultigen in the early Holocene, primary domestication of the crop may have been earlier than this in South to Southeast Asia (during the Pleistocene), within the western part of the natural range. Their work pointed to the origin of tropically cultivated taro (and a widespread form of commensal wild taro) in the general vicinity of the Bay of Bengal.

Approaches to defining the natural range of taro (Matthews, 1991, 2014, 2023; Matthews et al., 2017) are contingent on how the species is delimited by taxonomists. It is also important to recognize that commensal wild taros (i.e., those associated with human settlement) can have multiple origins as invasive natural wildtypes, as useful wild plants that have naturalized after being transplanted without cultivation, and as feral, self‐dispersing garden escapes. Currently, taro is known as a highly polymorphic species with two common cultivated morphotypes and many intermediate forms (Ahmed et al., 2020; Hay, 1998; Lakhanpaul et al., 2003; Orchard, 2006; Plucknett, 1983). Vegetative and floral traits found in the diverse cultivated forms can be found in multiple wild *Colocasia* species, making it difficult to include *C. esculenta* in taxonomic keys for wild species (Matthews et al., 2022). Taxonomically, the definition of taro has always been difficult (Hay, 1998), in part because the first formal botanical descriptions were based on cultivars and in part because earlier descriptions employ fewer characters than later descriptions, making comparison difficult. Taxonomic uncertainty may also reflect intra‐specific hybridization within taro and interspecific hybridization between taro and other *Colocasia* species (Ahmed et al., 2020; Matthews, 2014, 2023).

Chloroplast DNA in taro was first studied using analysis of restriction enzyme fragment polymorphisms (RFLPs) in wild and cultivated taros from across Asia (Ochiai et al., 2000; Yoshino, 1994). Since then, partial and complete chloroplast genome sequences have been published for many genera of Araceae, including *Colocasia* (Abdullah, Henriquez, Mehmood, et al., 2021; Ahmed et al., 2012, 2013, 2020; Henriquez et al., 2014; Ly et al., 2017; Nauheimer et al., 2012; Nauheimer & Boyce, 2014). Uniparental, maternal transmission of chloroplast genomes in taro has not yet been demonstrated, but is likely as this pattern is usual in most angiosperms (Greiner et al., 2015). Agricultural researchers have mostly studied the relatively narrow genetic diversity present in collections of clonally propagated taro cultivars, using analysis of simple sequence repeats (SSRs) or genome‐wide single nucleotide polymorphisms (SNPs), for example (Bammite et al., 2021; Chaïr et al., 2016; Helmkampf et al., 2017; Wang et al., 2020). Near‐complete nuclear genome sequences have been reported for a small number of taro cultivars (Bellinger et al., 2020; Soulard et al., 2017; Yin et al., 2021), and nuclear DNA sequence data have been generated for 128 species in 111 genera of Araceae, including taro, using target sequence capture and the Angiosperms 353 universal probe set (Haigh et al., 2022). These recent studies have produced reference data sets that raise many new possibilities for evolutionary studies of taro and its wild relatives.

Together with a wide geographical survey of wild and cultivated taros, Ahmed et al. (2020) presented the first draft of a chloroplast phylogenetic tree for taro, revealing three main haplotype groups or lineages (“clades” CI–III). This tree was based on the sequences of six selected chloroplast loci (Ahmed et al., 2013), two *Colocasia* species, *C. esculenta*, and *Colocasia formosana* (Hayata, 1919; Matthews et al., 2015), and two distant‐outgroup aroid genera (*Remusatia* and *Steudnera*). Ahmed et al. (2020) found one especially widespread haplotype (C1, Type 1) in tropical, cultivated taros (in Africa, Asia, and Oceania) and commensal wild taros (Southeast Asia to southern Japan).

Here, we extend the genealogical tree structure by analysis of complete chloroplast genome sequences in nine additional samples of *C. esculenta*, and eight additional samples from five other *Colocasia* species: *Colocasia fallax* Schott (Ara, 2000; Deva & Naithani, 1985), *C. formosana*, *Colocasia lihengieae* (Long & Liu, 2001), *Colocasia oresbia* (Hay, 1996), and *Colocasia spongifolia* (Matthews et al., 2022). The results indicate that within *Colocasia*, *C. fallax* is distant from and *C. oresbia* is near a group comprised of *C. formosana*, *C. lihengieae*, *C. spongifolia*, and *C. esculenta*. Our analysis thus included a larger number of close relatives of taro (three versus one) than previously reported (Ahmed et al., 2020), and produced a phylogenetic tree supported by high non‐parametric bootstrap values. We show that the three chloroplast lineages within *C. esculenta* are not close sisters and that the close wild relative *C. formosana* is distinct from *C. esculenta*. We also show that the CI and CII lineages form a close sister group that includes cultivated taros with morphotypes generally known as dasheen (“var. *esculenta*”, producing large mother corms) and eddoe (“var. *antiquorum*”, producing many side corms). The CIII lineage of wild *C. esculenta* is not closest to CI and CII. Rather, it appears closest to *C. lihengieae* within the CIII haplogroup, which also included *C. formosana* and *C. spongifolia*. Wild taros, generally known as *C. esculenta* “var. *aquatilis*” (producing long stolons with indeterminate growth and being semi‐aquatic) were represented by CI *and* CIII haplotypes.

So far, the CIII lineage has been found in wild *C. esculenta* and three other, wild *Colocasia* species (Ahmed et al., 2020, and present results). To explain the grouping of CIII wild taros (*C. esculenta*) with other wild *Colocasia* species, we suggest that *C. esculenta* and an unknown parent species (*Colocasia* sp.) were involved in a process of hybridization, chloroplast capture, and range extension. The results also point to the Assam‐Bengal floodplain as a region of key interest for exploring the complex genetic and geographical origins of tropical cultivated taros.

## 2 MATERIALS AND METHODS

**FIGURE 1:**
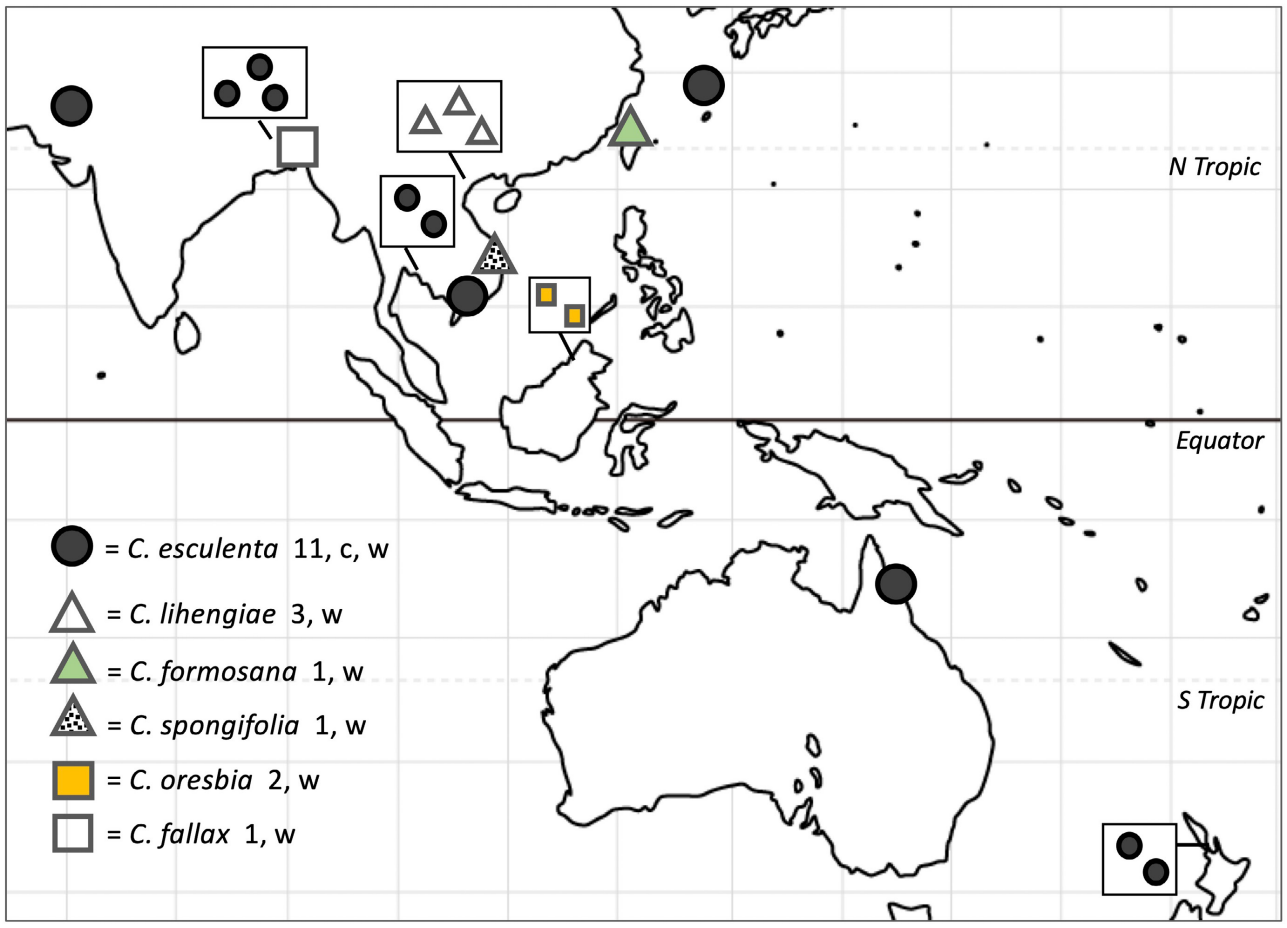
*Colocasia* species sampled. Key shows: Species name, number of samples sequenced (total = 19), c = cultivated, w = wild. Circles = *C. esculenta*; triangles = closely related species, squares = outgroup taxa. Multiple samples from within a limited area are shown inside boxes (schematic). The *C. lihengieae* group includes a possible hybrid. Sample details are given in Table A1.

**FIGURE A1:**
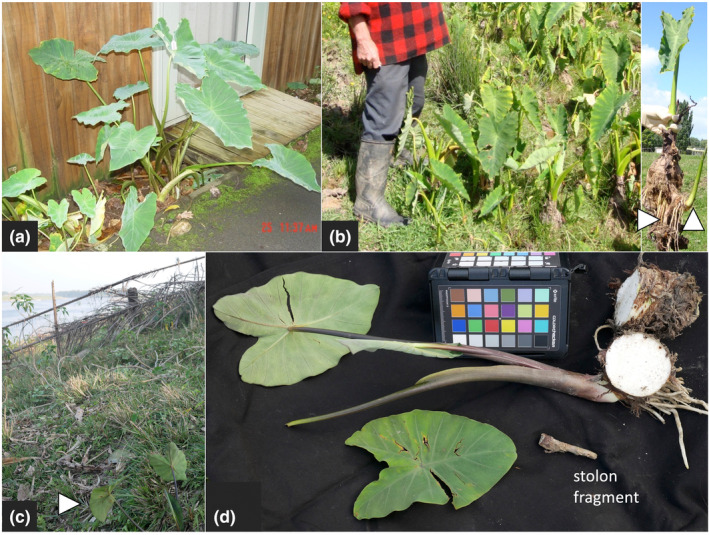
*Colocasia esculenta* (CI) in New Zealand and Bangladesh. (a, b) var. GP (Matthews, 1985, 2014). (a) CESNZ03 (*ex horto*); sampled plant in campus garden, University of Auckland, New Zealand; photo by I. Ahmed, 25th June 2008. (b) Typical commensal wild, clonal population in wet ground, surrounded by pasture, and single large corm (at right) at least 4 years old based on visible record of seasonal changes in corm diameter – with stolon (lower arrow) and directly‐growing side‐shoot (upper arrow); vicinity Whangarei, northern New Zealand; photos by P. J. Matthews, 29th October 2009. (c, d) CESBD01, commensal wild taro (with large starchy corm, so likely an escaped cultivar) on grazed river bank, Mymensingh, Bangladesh; photos by P. J. Matthews, 10th Feb. 2019. (c) Habitat and single plant (arrow) found and sampled. (d) Whole plant with starchy corm and stolon fragment.

**FIGURE A2:**
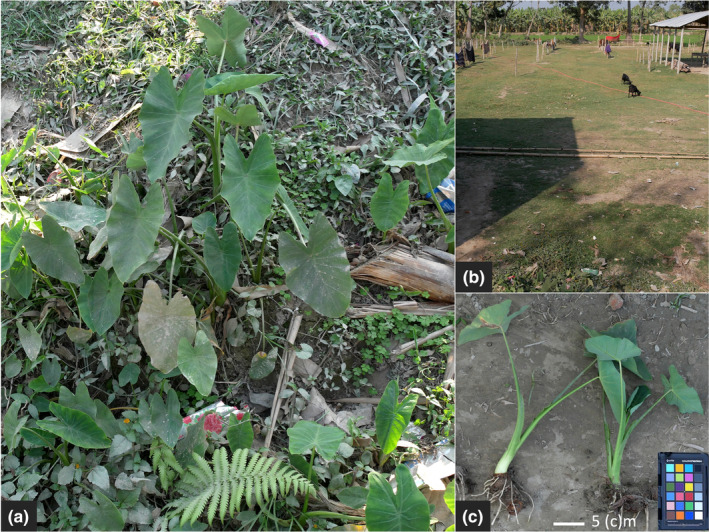
*Colocasia esculenta* (CI), CESBD02, c.f. var. *aquatilis* (Matthews 1991, 2014; Matthews et al., 1992), commensal wild plant, in grazed field, Sylhet, Bangladesh. (a) Habit; clump spreading with stolons. (b) Habitat, showing adjacent field with cattle and goats. (c) Sampled plant, excavated to show morphology; leaf all green, blade oval to angular sagittate, margin undulating, with deep sinus; 6–8 primary lateral veins (side‐ribs) branching on each side from costae (main‐ribs) of the posterior and anterior lobes (smallest leaves have fewer side‐ribs). Photos: P. J. Matthews, 13th Feb. 2019.

**FIGURE A3:**
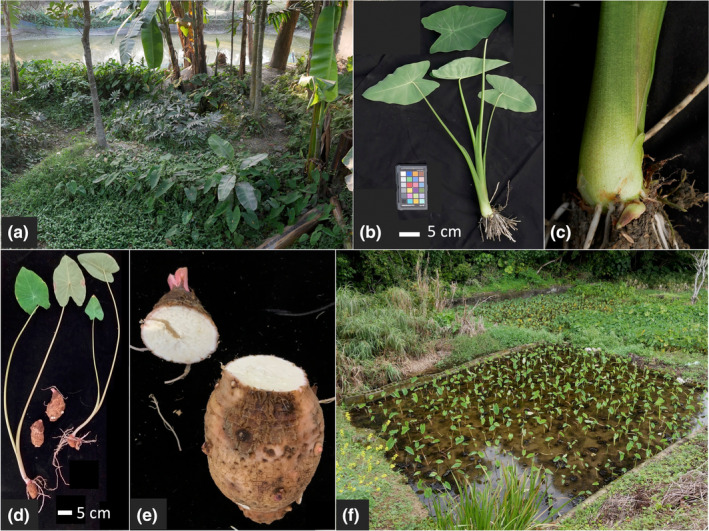
*Colocasia esculenta* (CI, wild and cultivated) in Bangladesh and Japan. (a–c) CESBD03, commensal wild taro next to fish pond, Sylhet, Bangladesh; photos by P. J. Matthews 13th Feb. 2019. (a) Habitat. (b) Whole plant; leaf all green with angular sagittate blade, wide and deep sinus, pale petiole base, and white roots. (c) Detail to show sprouting stolon, lower right with purplish tip (c.f. var. *aquatilis*, Matthews 1991, 2014; Matthews et al., 1992). (d–f) Cultivar *Ta‐imo*, Kikai island, southern Japan; photos by P. J. Matthews. (d) CESJP30, sampled plant, kept in pot ex situ. (e) detail showing starchy corm, pink petiole base and pink buds (July 2022). (f) pondfield with *Ta‐imo* (14th March 2017).

**FIGURE A4:**
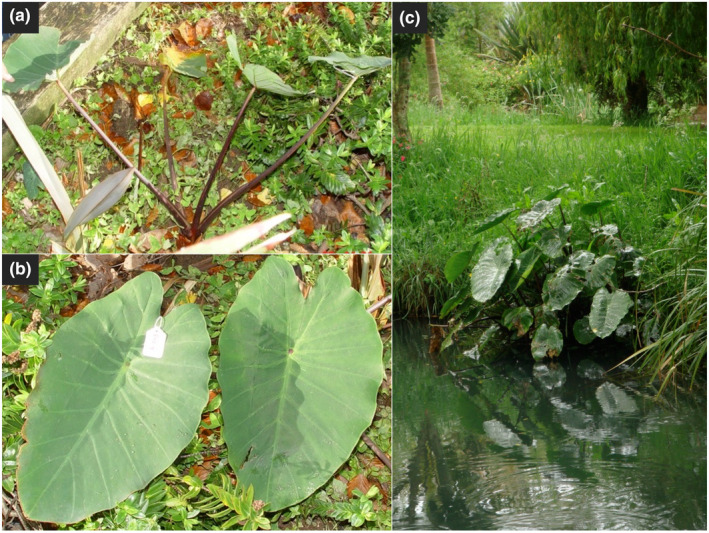
*Colocasia esculenta* var. RR (lineage CII, triploid cultivar and commonly a commensal wild plant in New Zealand) (Matthews, 1985, 2014). (a, b) CESNZ02 (*ex horto*); sampled plant in campus garden, University of Auckland, New Zealand; photos by I. Ahmed, 25th June 2008 (chloroplast sequence reported in Ahmed et al., 2020). (c) Commensal, wild plants in a clonal clump on stream bank next to pasture (in heavy rain, hence wet leaves despite waxy surface); Pakaraka, Bay of Islands, northern New Zealand. Photo: P. J. Matthews, 26th October 2009.

**FIGURE A5:**
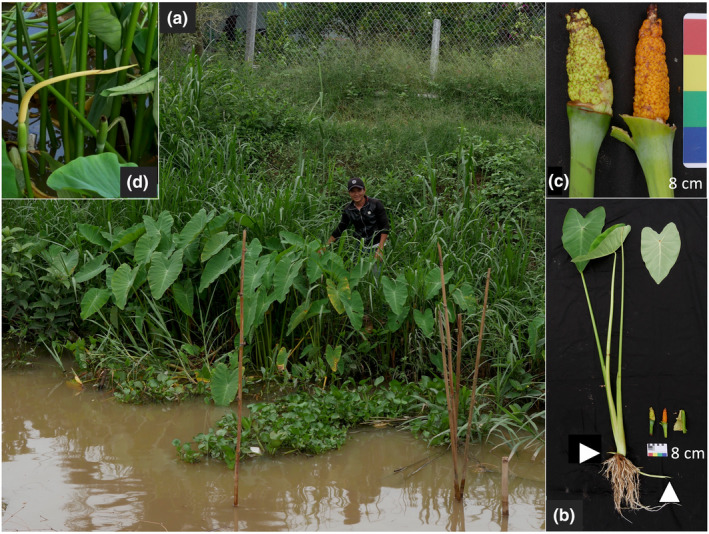
*Colocasia esculenta* (CIII wild taro), c.f. var. *aquatilis* (Matthews, 1991, 2014; Matthews et al., 1992). (a–c) CESVN15, commensal wild plants on canal bank; delta island between the main Mekong river and Song Hau, Mekong, Vietnam. (a) Habitat and habit (clump spreading with long stolons). (b) Sampled plant showing all‐green leaf, corm with young stolons (see arrows), and fruiting heads collected at the same site. (c) Detail showing the near‐mature (green) and fully‐mature (orange) fruiting heads; note oval to angular, sagittate blade with wide and deep sinus, and 8–10 primary lateral veins (side‐ribs) branching on each side from costae (main‐ribs) of the posterior and anterior lobes. (d) Detail showing inflorescence with yellow upper spathe much longer than the spadix, seen at a nearby site (WP733) with similar plants, at edge of Mekong river, main channel. Photos: P. J. Matthews, 21st Sept. 2017.

**FIGURE A6:**
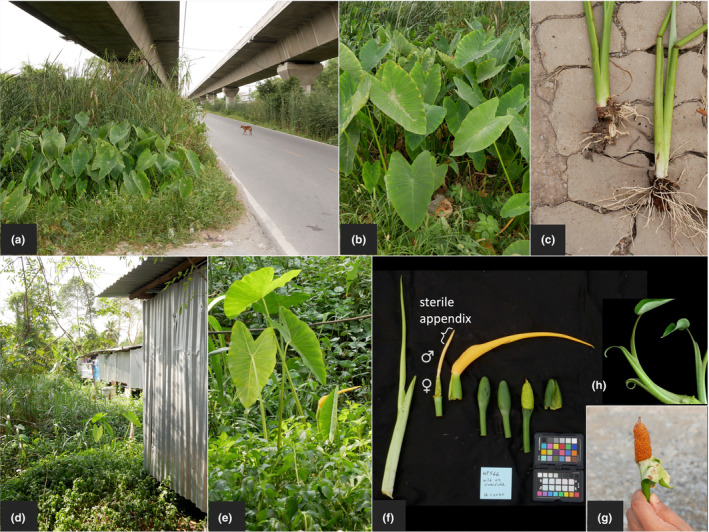
*Colocasia esculenta* (CIII wild), c.f. var. *aquatilis* (Matthews, 1991, 2014; Matthews et al., 1992). (a–c) CESTH06 collection site; plants wild in vacant land (former farmland) under motorway, Khlong Prapa, Bangkok, Thailand; photos by P. J. Matthews, 18th Feb. 2019. (a) Sampled roadside population, (clump spreading with long stolons). (b) Detail showing upper leaf morphology. (c) Detail showing green petioles, white at base with white basal ring; corms with white roots, skin, cortex, core parenchyma; note slightly angular, sagittate blade with wide and deep sinus, and 8–10 primary lateral veins branching on each side from costae of the posterior and anterior lobes. (d, e) CESTH07 collection site; commensal wild plants, Ko Kret Island, Chao Praya, Bangkok, Thailand; photos by P. J. Matthews, 18th Feb. 2019. (d) Sampled population in swamp inland behind houses built near shore of river island. (e) Detail showing blades, and long, upper spathe curving to near horizontal position. (f–h) Reproductive morphology at other sites, typical of the wild populations in and around Bangkok, photos by P. J. Matthews, 16th–17th Jan. 2020, and ex situ 27th Aug. 2022. (f) Mature inflorescence, and series of fruiting heads from the same plant; sterile appendix more than half the length of male zone (here ca. 0.7×; at other sites equal), and yellow upper spathe much longer than spadix, and nearly horizontal. (g) Ripe fruiting head with soft, orange berries. (h) Tip of young stolon with tendrils (potted plant, ex situ).

**FIGURE A7:**
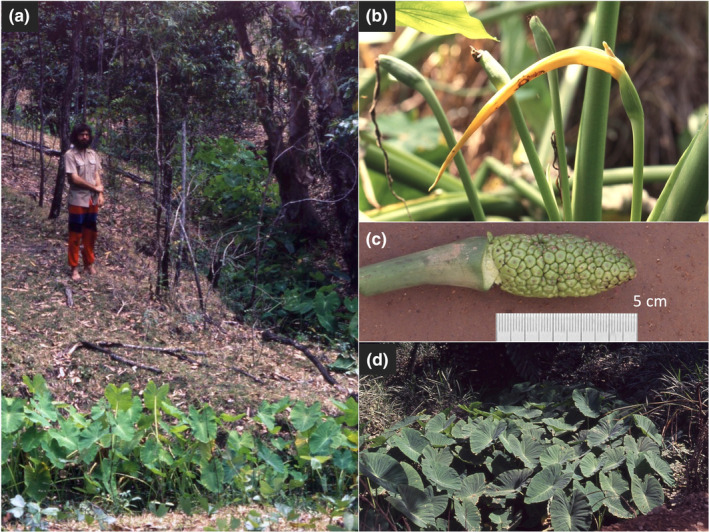
*Colocasia esculenta* var. *aquatilis* in Australia (CIII wild) (Matthews, 1991, 2014; c.f. Matthews et al., 1992). (a) CESAU24 collection site with wild taro population in riverine forest, at Hopevale, northeast Queensland, Australia (photo by K. Thiele, 26th Sept. 1987; Matthews at left). (b–d) Wild taros, Cape Tribulation, northeast Queensland; photos by P. J. Matthews, mid‐Aug. 1992. (b) Mature inflorescence, and young fruiting heads; note short sterile appendix (less than half the length of male zone silhouetted in this image), and upper yellow spathe that is much longer than the spadix. (c) Near‐mature fruiting head, fully fertilised (all berries developed, with mature seeds inside, indicating effective insect pollination). (d) Population in wet gully; note oval‐sagittate blade with wide and deep sinus, and 8–10 primary lateral veins branching on each side from costae of the posterior and anterior lobes.

**FIGURE A8:**
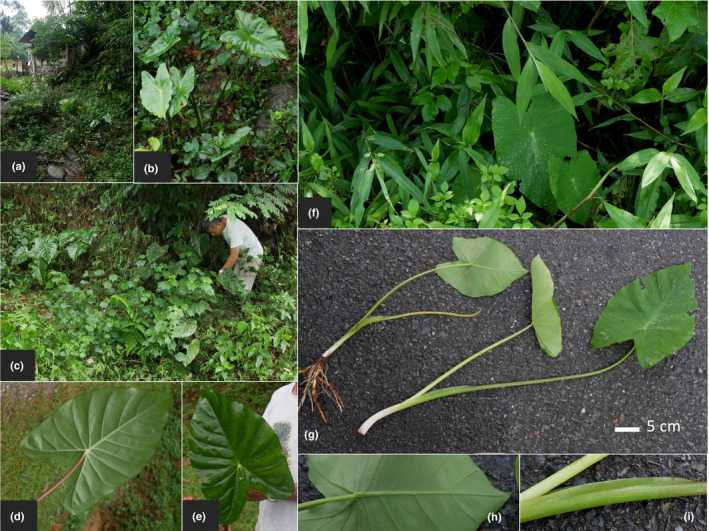
*Colocasia lihengiae* (Long & Liu, 2001) and a possible hybrid (all samples CIII). (a–e) (*C. lihengiae*). (a, b) CLIVN04 collection site with commensal wild population, at edge of forest, Phu Tho prov., Vietnam; photos: P. J. Matthews, 4th Oct. 2017. (c–e) CLIVN05 collection site, Tukuk Commune, Dang Son district, Vietnam; photos by P. J. Matthews, 4th Oct. 2017. (c) Plants wild on roadside bank, at edge of forest. (d) sampled plant showing petiole (purple) and lower side of blade. (e) Upper side of blade; note angular shape, apiculate apex, and shining surface (wettable, non‐waxy). (f–i) CxVN01 collection site with possible hybrid (*C. lihengiae* × *?menglaensis*); isolated plant wild on roadside bank, at edge of forest near Yen Bai/Phu Tho border, Vietnam; photos by P. J. Matthews, 5th Oct. 2017. (f) Sampled plant, with water adhering to leaf after splash test though leaf surface is not shiny (like *C. menglaensis*, and in contrast to wettable shiny leaf of *C. lihengieae*). (g) Same plant showing stolons, and angular blade with wide sinus and apiculate apex (shape similar to *C. lihengiae*). (h) Detail showing strongly raised secondary veins on lower surface between primary lateral veins (as in *C. menglaensis*). (i) Detail showing broad petiole sheath (as in *C. menglaensis*), but with smooth petiole surface (as in *C. lihengieae*, in contrast to hairy in *C. menglaensis*).

**FIGURE A9:**
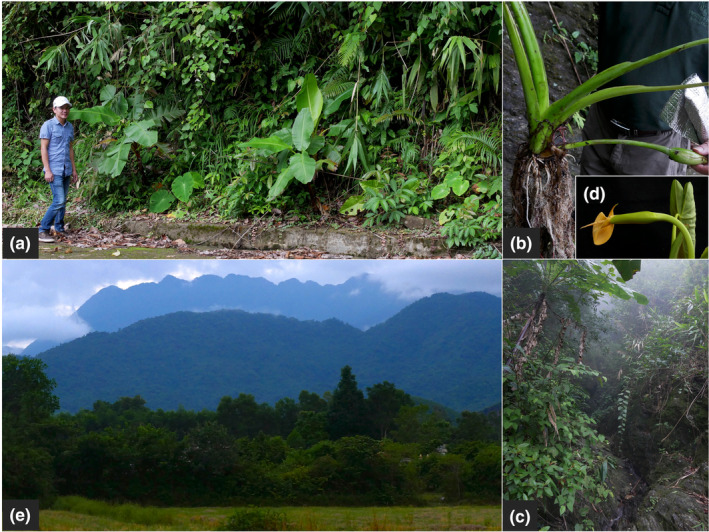
*Colocasia spongifolia* (Matthews et al., 2022). (a–c) CSFVN01 collection site, Bach Ma, Vietnam; photos by P. J. Matthews, 22nd Sept. 2018. (a) Scattered individual plants at roadside and edge of forest. (b) Fruiting plant from rock crevice. (c) View into adjacent cloud forest. (d) Inflorescence on seedling produced by seed from CSFVN01, growing in pot ex situ; photo by P. J. Matthews, Osaka, 29th July 2020. (e) View of seaward, terminal ridge of Bach Ma mountain range (partly obscured by low clouds) where the collection site is located; photo by P. J. Matthews, 22nd Sept. 2018.

**FIGURE A10:**
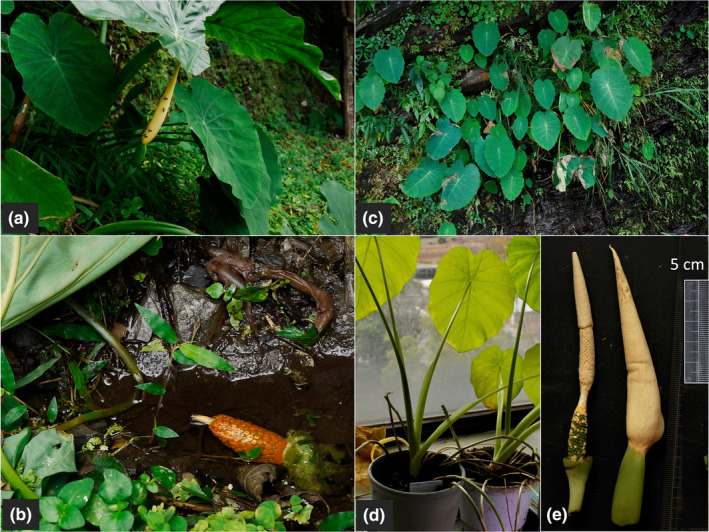
*Colocasia formosana* (Hayata, 1919, Hsu et al., 2000). (a–c) CFOTW03 collection site; plants wild on roadside at edge of forest, Wutai district, Pingtung County, Taiwan, photos by P. J. Matthews, 1st Sept. 2014. (a) Inflorescence with stingless bees on outside (and pollinating flies, *Colocasiomyia* sp., inside) (both insects are commonly seen on inflorescences in Taiwan). (b) Plants on wet rock face nearby, showing typical entirely green leaf, with rounded blade and shallow sinus. (c) Fully ripe fruiting head (reddish orange colour) after separation from peduncle and falling; seeds were collected. (d, e) Seedlings grown ex situ in Osaka; photos by P. J. Matthews. (d) Plant with vigorous stolons, 17th June 2015. (e) Inflorescence with large sterile appendix (longer than male zone, and base wider than male zone), and abundant sterile male flowers (staminodes, creamy yellow) among the female flowers, 11th Dec. 2018.

**FIGURE A11:**
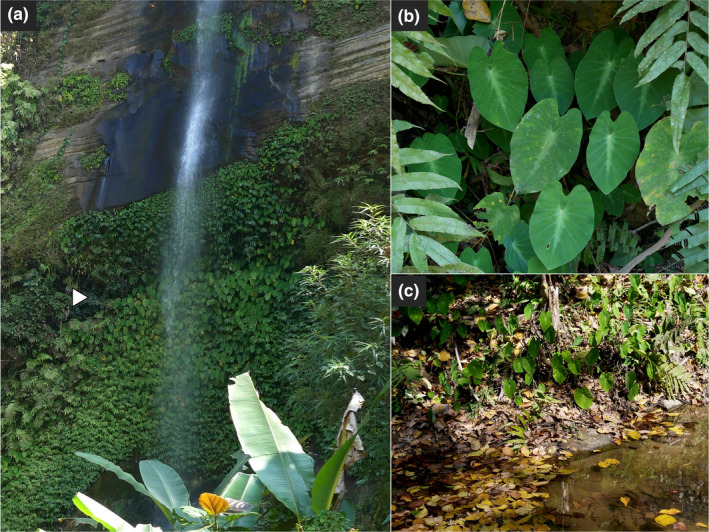
*Colocasia fallax* Schott (Ara, 2000; Deva & Naithani 1985). CFABD01 collection site with wild population, Madhabkunda, Bangladesh. (a) Large group of plants (to right of arrow) alongside *Elatostema* sp., on wet rock face below waterfall. (b) Plants on stream bank next to waterfall, with pale blush in blade centre. (c) Small group downstream on sandy stream bank. Photos: P. J. Matthews, 13th Feb. 2019.

All samples were collected by the present authors in locations across Asia and the Pacific (Figure 1, Table A1, and Figures A1, A2, A3, A4, A5, A6, A7, A8, A9, A10, A11). Previous studies (Ahmed et al., 2013, 2020) identified specific chloroplast loci and PCR primers that can be used to identify haplotypes. We used this approach to test samples in pilot surveys of wild taro populations and wild *Colocasia* species in Bangladesh, Thailand, and Vietnam, identify CI and CIII individuals, and then choose samples of *C. esculenta*, *C. formosana*, *C. lihengiae*, and *C. spongifolia* for complete genome sequencing in order to represent as much haplotype diversity as possible within our budget. One cultivar with small side corms, from a market in northern Pakistan, was included without prior testing and provided a new CII sequence.

For recently collected samples, DNA was extracted using the protocol of Ahmed et al. (2009) with minor modifications. The oldest sample, from Australia, was collected in 1987, extracted as described by Matthews (2014), and preserved in a frozen DNA archive. Seventeen samples were newly sequenced using the Illumina PE 150 run (Genwiz Life Sciences, China). Bioinformatic analyses, including sequencing‐data quality checks, genome assembly, annotations, circularization, and data curation, were done as reported previously (Abdullah, Henriquez, Mehmood, et al., 2021). Subsequent analyses included alignments of two previously reported complete chloroplast genome sequences (Ahmed et al., 2012) from New Zealand (CESNZ03, var. GP, triploid, and var. RR, triploid, CESNZ02; Table A1) and the 17 new samples (see Data Availability Statement). Using MAFFT in Geneious, sequence sets (see below) were aligned, and one copy of the inverted repeat (IRa) and all gaps (indels) were removed. Removal of IRa is required to avoid double representation of the large repeat region. The online software IQ‐Tree (Nguyen et al., 2015) was used to perform Maximum Likelihood (ML) analysis with “auto” model selection and bootstrapping for 1000 times in Ultrafast mode (bootstrapping 100 times in Standard mode gave similar results). Outgroups were automatically inferred by IQTree. Model selection by IQ‐Tree employed ModelFinder (Kalyaanamoorthy et al., 2017). Tree diagrams produced by IQ‐Tree were prepared for presentation using the “branch‐swapping” functions of TreeDyn (Chevenet et al., 2006).

**FIGURE 2:**
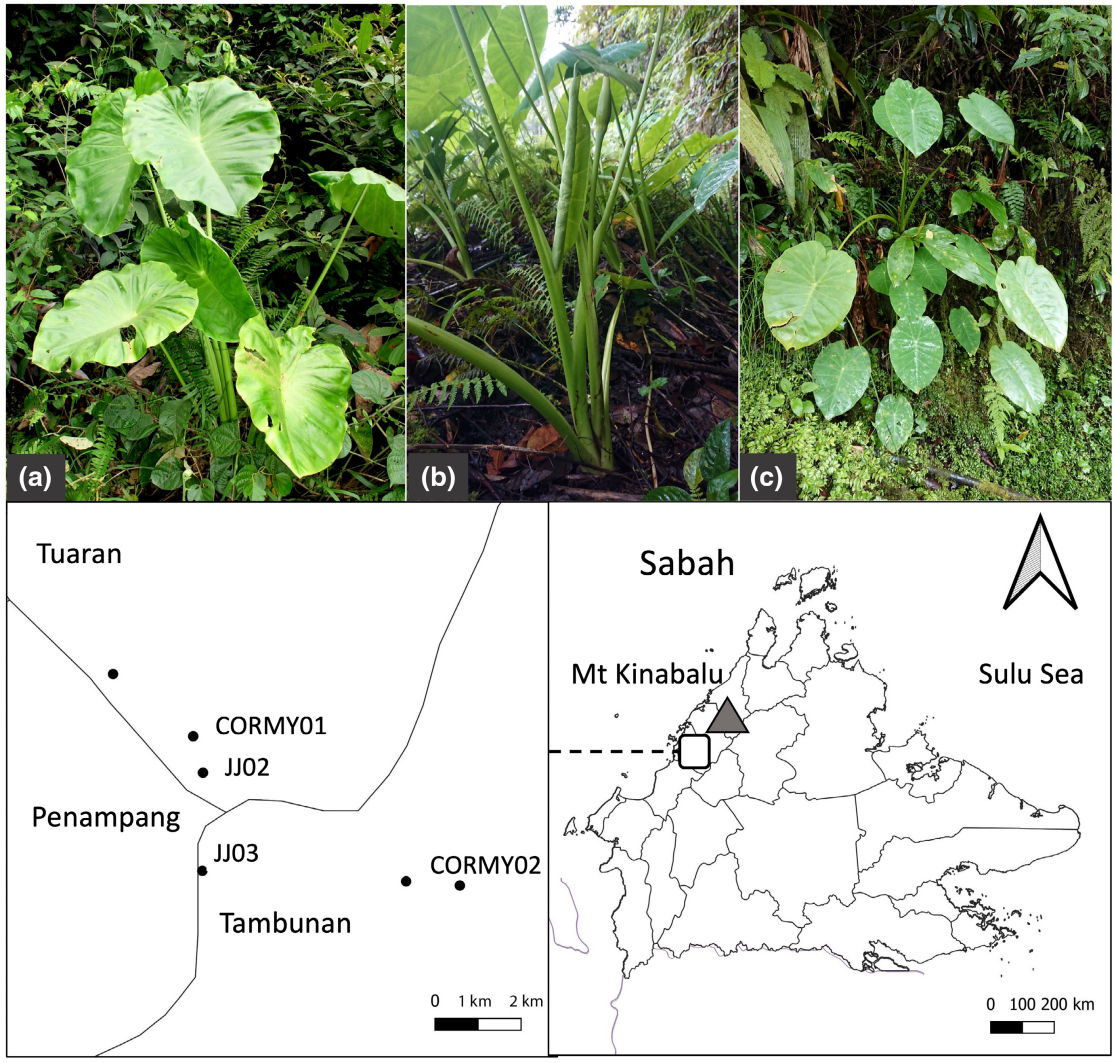
*Colocasia oresbia* (Hay, 1996) in Borneo, Malaysia: Plant habit and locations. *C. oresbia* has an erect habit, lacks stolons, and does not form spreading clumps: (a) Plant in full sun, Nanga Gaat, Sarawak (photo: P. Boyce, 13th May 2004). Sabah locations (photos: J. Joling, 22nd Dec. 2020): (b) JJ02 fruiting in semi‐shade, (c) JJ03 in semi‐shade. Map, right: Type location for *C. oresbia* on Mt Kinabalu (triangle). Map detail, left: Source locations for sequenced samples (CORMY01, CORMY02) in a scattered population of mostly isolated plants (black dots) in Sabah. District names and boundaries are shown. Sample details are given in Table A1.

**FIGURE A12:**
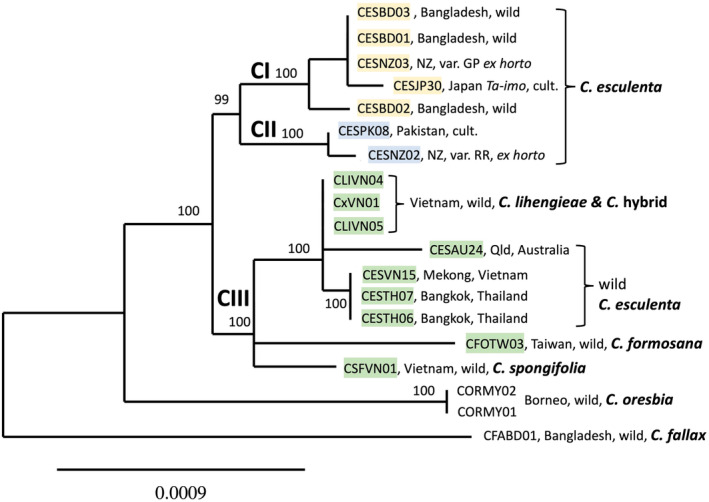
ML tree with *C. fallax* and five other *Colocasia* species, based on all protein‐coding gene sequences (CDS), excluding those of IRa (one of two copies of the large inverted repeat). *C. fallax* is placed as a distant outgroup, and *C. oresbia* is placed as a near outgroup for *C. esculenta* and its close wild relatives. Sample details are given Table A1.

Initial ML analysis using an alignment of all protein coding sequences (CDS, total concatenated, aligned sequence of 68,985 bp) from all available *Colocasia* species placed *C. fallax* as a distant outgroup and *C. oresbia* (Figure 2) as a near outgroup for *C. esculenta* and closely related taxa (Figure A12, Method details in Appendix A). We therefore excluded *C. fallax* from further sample sets to avoid (a) long‐branch attraction bias in the phylogenetic tree (reviewed in Bergsten, 2005), and (b) information loss from insertion/deletion sites that may appear when target ingroup sequences are aligned with a distant outgroup.

For analysis of *C. oresbia* (the near outgroup), *C. esculenta*, and close relatives, complete genome sequences were aligned, giving a total aligned sequence of 134,382 bp with 696 parsimony‐informative sites. To improve subclade resolution, all intergenic sequences from *C. esculenta* and close relatives (with *C. oresbia* excluded) were aligned, giving a total aligned sequence of 46,269 bp with 249 parsimony‐informative sites.

To compare bootstrap support in relation to the density of parsimony informative sites across different sequence partitions (CDS, complete genome, intergenic), for each sample set, we calculated the average bootstrap value across all nodes in each ML tree (average BS percentage) and used log data from IQ‐Tree runs to calculate the number of parsimony informative sites per 100 bp of aligned sequence (average PI). To gain an indication of the impact of model misspecification on these calculated parameters, we also compared trees built with models selected by ModelFinder and trees built with the Jukes‐Cantor model and ML optimization in IQ‐Tree. These calculations and comparisons are reported in Method details in Appendix A and discussed in relation to methodological constraints in Discussion section in Appendix A.

## 3 RESULTS

**FIGURE 3:**
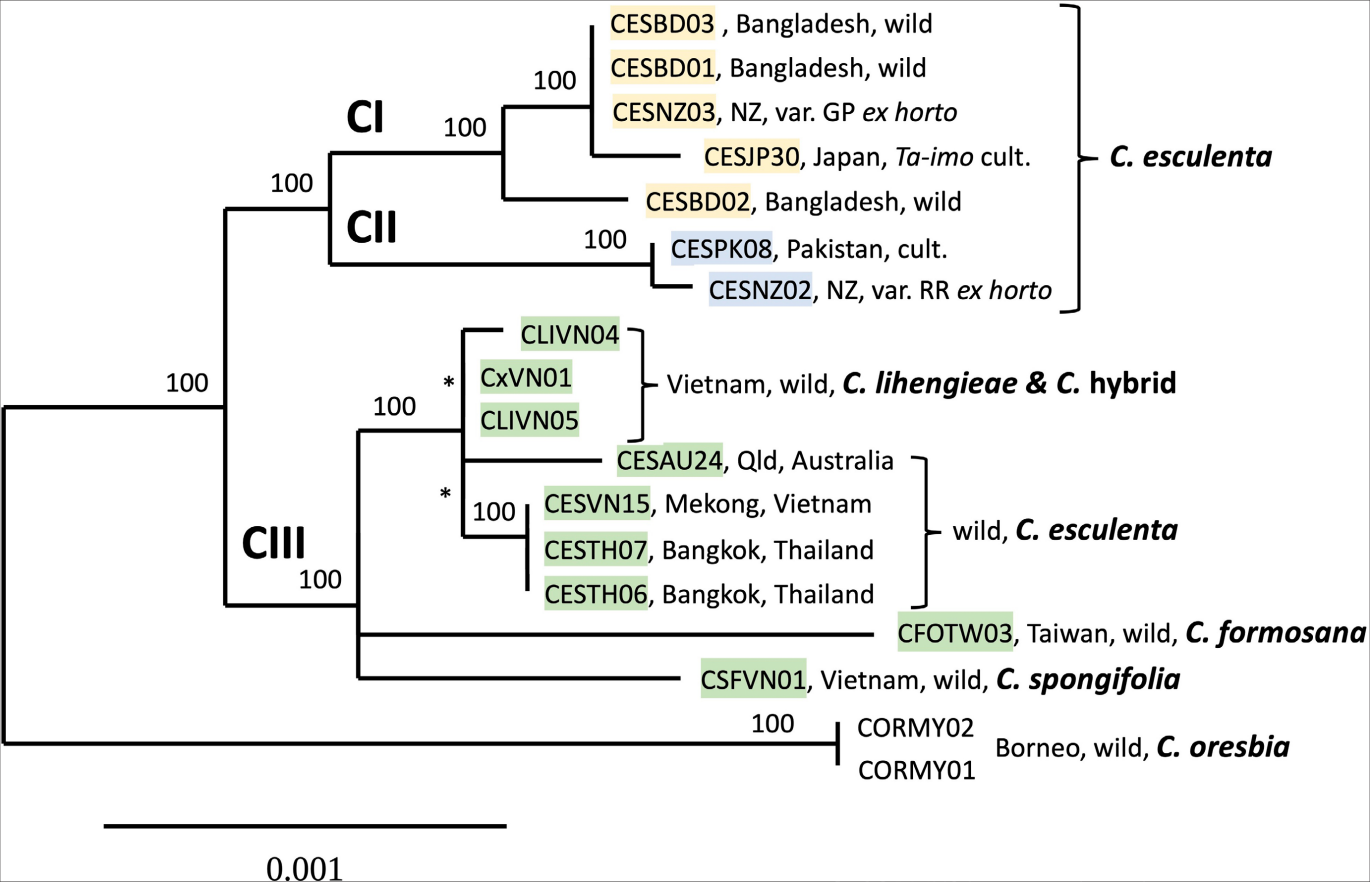
ML tree for *C. esculenta* and close relatives, including *C. oresbia* (identified as a near outgroup through preliminary analysis) and based on complete chloroplast genome alignment. * = node with 100% support next to very short (indistinct) branch. The scale for branch lengths (lower left) indicates number of substitutions per nucleotide position (divergence). The colour coding for main lineages is used throughout this paper: CI (pale orange), CI (blue) CIII (green). Sample details are given Table A1.

**FIGURE 4:**
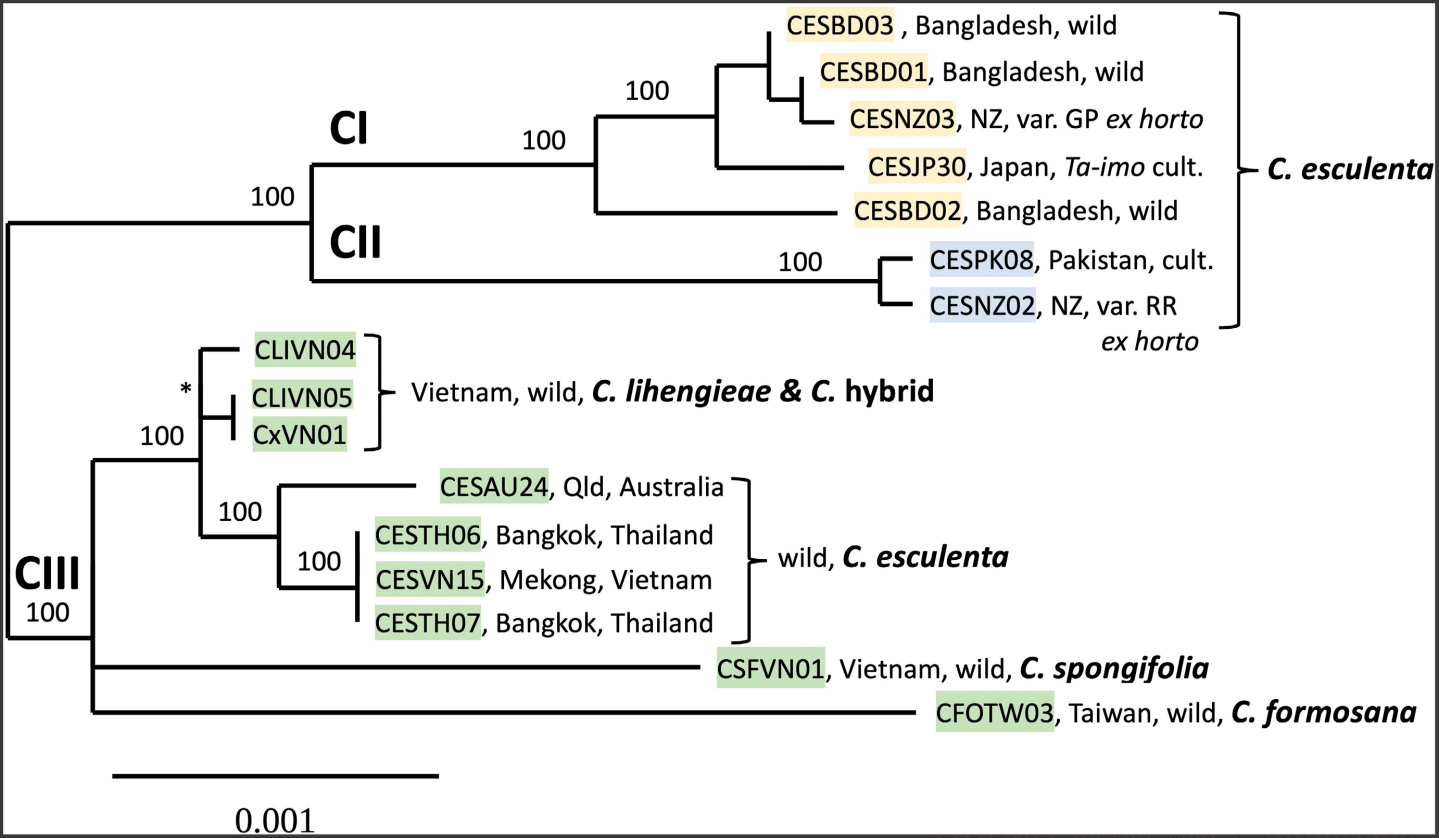
ML tree for *C. esculenta* and close relatives, excluding the near outgroup *C. oresbia* and based on total intergenic sequence (IGS) alignment. Sample details are given Table A1.

Our initial, more inclusive analysis placed *C. fallax* as distant from all the other species (Figure A12). Subsequent analyses without *C. fallax* allowed alignment of the remaining sequences with fewer gaps and provided a well‐supported genealogical tree structure (phylogenetic hypothesis), with *C. oresbia* standing as a near outgroup and *C. esculenta* (taro) grouped together with *C. lihengiae*, *C. formosana*, and *C. spongifolia* (Figure 3). Two chloroplast lineages, previously identified as clades CI and CII in *C. esculenta* (Ahmed et al., 2020), form a well‐defined group of sister lineages with a deep split. These are found in cultivated taros known as var. *antiquorum* (eddoe, CII), var. *esculenta* (dasheen, CI), and in a widespread, commensal wild form known as var. *aquatilis* (CI). A further group of wild taros (also var. *aquatilis*) from Australia, Vietnam, and Thailand form a distinct subgroup within the CIII haplogroup alongside the wild species *C. lihengiae*, *C. formosana*, and *C. spongifolia* (Figure 4), separate from the CI and CII sister lineages. The apparent non‐congruence between chloroplast lineages (haplogroups) and morphological diversity within *C. esculenta* is summarized in Table 1 (a schema based on present results and the findings of Ahmed et al., 2020).

**TABLE 1:** Schema showing non‐congruencies between morphotype and chloroplast genome haplogroup in taro and closely related species: var. *aquatilis* is associated with the CI and CIII haplogroups, while CI is associated with the “dasheen” and var. *aquatilis* morphotypes, and CIII is associated with the non‐stolon‐producing morphotype in *C. spongifolia* and stolon‐producing morphotypes in other species.

Haplogroups	*Colocasia esculenta*			*Colocasia* sp.
	var. *esculenta* (“dasheen”, with large mother corm)	var. *antiquorum* (“eddoe”, with many side‐corms)	var. *aquatilis* ^a^ (long stolons)	*C. formosana* ^a^, *C. lihengiae* ^a^, *C. spongifolia* ^b^
CI	+	−	+	−
CII	−	+	−	−
CIII	−	−	+	+

^a^ Produces long stolons.

^b^ Shy‐sprouting; new shoots sprout directly from erect or decumbent mother stem.

Complete chloroplast genome sizes ranged from 161,252 bp in *C. fallax* to 161,973 bp in *C. oresbia* and 162,644 bp in *C. esculenta* (Table A1). The overall genome structure was the same in each sample and similar to previous reports for *C. esculenta* (Abdullah, Henriquez, Croat, et al., 2021; Abdullah, Henriquez, Mehmood, et al., 2021; Ahmed et al., 2012). With *C. fallax* excluded and using a complete chloroplast genome alignment, there was clear resolution of tree structure within *C. esculenta* and closely related taxa (Figure 3), with PI = 0.518 sites/100 bp and an average BS value = 93.9%. With *C. oresbia* excluded and using intergenic sequences only, there was better resolution of branches within CI and CIII (Figure 4) and a similarly robust result: PI = 0.538 sites/100 bp and average BS value = 94.8%.

## 4 DISCUSSION

The main findings of interest for the evolution of taro (*C. esculenta*) are the non‐congruence between chloroplast genome diversity and morphological diversity within the species and the grouping of CIII wild taro with other wild *Colocasia* species and *C. lihengiae* in particular (Figures 3 and 4, Table 1). Later, we propose three different evolutionary scenarios that may explain these findings. Note that the schema shown in Table 1 requires further substantiation with DNA tests on a wider range of cultivated samples of known morphology, and that cultivated taros might include hybrids that contradict the present schema. The ML tree in Figure 4 has apparently better resolution of CIII wild taros as a distinct subgroup, a result obtained by excluding *C. oresbia* and analysing just the intergenic sequences, which have a higher density of parsimony informative sites than other genome partitions (Table A2). The labels CI–CIII were previously used to identify “clades” (Ahmed et al., 2020) but are used here to identify haplogroups or lineages. The term “clade” can be understood to represent a group of taxonomic species with a shared common ancestor, while our data are primarily a representation of diversity among individual chloroplast genomes (haplotypes), from which we first attempt to make inferences about relationships among chloroplast haplogroups and then among taxonomic species. A possible *Colocasia* hybrid (sample CxVN01, Figure A8) was identified in the field as a possible hybrid of *C. lihengieae* and *C. menglaensis*, which are sympatric species in the northern mountains of Vietnam. Further studies of this sample and adjacent populations of *Colocasia* spp. are needed to gain insight into local gene flow and the host preferences of specialist insect pollinators (*Colocasiomyia* spp.) associated with *Colocasia* spp. (Takano et al., 2021).

Further results of interest (1–4) are:

1. *Colocasia oresbia* (Figures 2 and 3) appears to be a near outgroup and is expected to be a valuable reference taxon for future studies of *C. esculenta* and its close wild relatives. The habit and and floral structure of *C. fallax* is very distinct, and was expected to be distant from the other species studied here; it was included to help clarify the relationship between *C. esculenta* and *C. oresbia* (an island Southeast Asian isolate).
2. CIII haplotypes were absent in the wild Bangladesh taros tested and form a distinct lineage (Figure 4) that is distributed in wild taros (also var. *aquatilis*) from Vietnam to Thailand and northern Australia; natural dispersal of this lineage, from Southeast Asia to Australia and New Guinea, may have occurred during the late Miocene to late Pleiocene (Ahmed et al., 2020).
3. CI diversity in Bangladesh (Figures 3 and 4) includes a commensal wild plant (Figure A1b, likely derived from nearby cultivation) that is sister to var. GP, a widespread triploid clone in northern New Zealand (Figure A1a, and Matthews, 2014); this suggests that early (19th century?) British shipping to New Zealand might have introduced var. GP from the Bengal region.
4. *Ta‐imo* (Figures 3 and 4), a common pondfield cultivar in southern Japan (Figure A3), displays the CI Type 1 haplotype, the most widespread haplotype in Asia and the Pacific, in cultivated and commensal wild taros (Ahmed et al., 2020); since Type 1 is nested among the wild CI taros found in Bangladesh, it might also originate in the Bengal region (see further discussion below).

The results also have implications for taxonomy. Although CI and CII form a well‐defined sister group, the deep split might correspond to a species‐level evolutionary divergence between the progenitors of CII cultivars known as var. *antiquorum* (eddoe), and those of CI taros that include var. *esculenta* (dasheen) and widespread, commensal forms of var. *aquatilis* (Ahmed et al., 2020).

**FIGURE A14:**
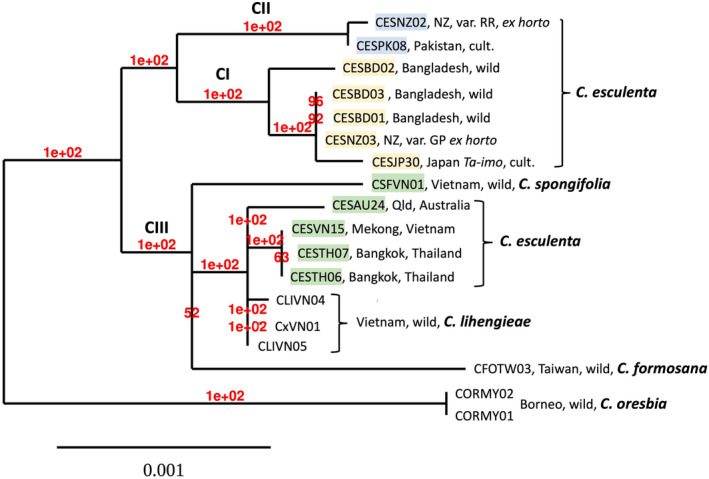
ML tree based on complete sequence data (all genic and intergenic regions, after removing IRa and gaps) from 18 samples (five species). Original IQ‐Tree output with bootstrap values. See final tree in Figure 3.

**FIGURE A15:**
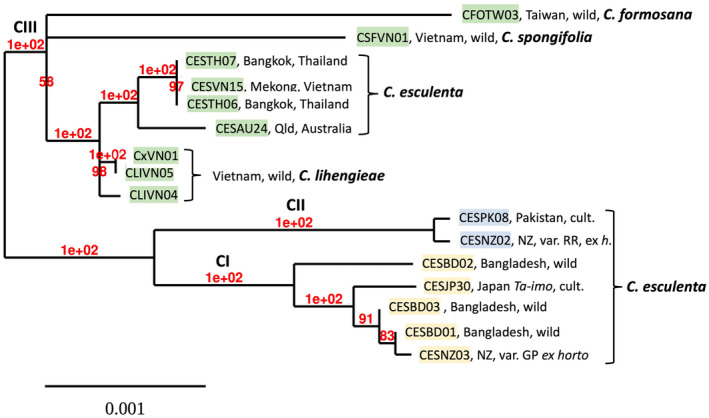
ML tree based on all intergenic sequence data (after removing IRa and gaps) from 16 samples (four species). Original IQ‐Tree output with bootstrap values. See final tree in Figure 4.

The CIII species group (*C. formosana*, *C. spongifolia*, *C. lihengiae*, and *C. esculenta*) includes an apparent polytomy (Figures 3 and 4) with weakly supported indistinct nodes (Figures A14 and A15). This might be an artefact of incomplete taxonomic sampling of closely related *Colocasia* species. Adding further species will probably resolve the polytomy. Within the group, *C. formosana* appears to be a distinct species and not a part (Li & Boyce, 2010) or an ecotype (Matthews et al., 2015) of *C. esculenta*. In wild breeding populations across Taiwan and in the northern Philippines, *C. formosana* is morphologically uniform and distinct from wild populations of *C. esculenta*.

According to a study of plants initially identified as *C. lihengiae* in North East India (Gogoi et al., 2019), the name *C. lihengiae* may be synonymous with *C. mannii* Hook.f. (Hooker, 1894). To confirm this, it will be useful to compare *C. mannii* in India with populations of *C. lihengiae* in Thailand (Sangnin, 2002), Vietnam (Nguyen et al., 2016), and southern China (Long & Liu, 2001). *Colocasia lihengiae* was placed in *C. antiquorum* (Schott, 1832) by Li and Boyce (2010), while *C. antiquorum* is usually placed within *C. esculenta* (Hay, 1998; Orchard, 2006). Our results suggest that *C. lihengiae* is close to but distinct from wild *C. esculenta* in the CIII species group (Figures 3 and 4). However, *C. lihengiae* is represented here by just three samples collected within a radius of 30 kilometres in northern Vietnam, including one apparent hybrid (CxVN01). As a widespread but little‐studied species, *C. lihengiae* may have considerable unrecognized phenotypic and genetic diversity.

Next, we propose three alternative evolutionary scenarios to explain (a) the lack of congruence between the chloroplast genome and morphological diversity in wild and cultivated forms of *C. esculenta* and (b) the finding that *C. lihengiae* is a close sister to a wild form of *C. esculenta* within the CIII lineage.

### 4.1 Scenario 1: Long separated species

In this scenario, distinct chloroplast lineages correspond to distinct, long‐separated species: CIII wild taro is a separate species from *C. esculenta* and derived from an ancestral population of *C. lihengiae* (the nearest sister taxon, on a short branch in Figures 3 and 4), or from an unknown common ancestor of CIII wild taro and *C. lihengiae*.

If this is true, the nuclear genome of CIII wild taro should be more similar to that of *C. lihengiae* than that of CI taro, despite the overall morphological similarity between CIII and CI wild taros identified as (or comparable to) “var. *aquatilis*” (Table A1; Figures A2, A3, A6, and A7; and details in *Scenario 3* below). A further contra‐indication for this scenario is previous plant breeding research in the Pacific, which showed that a wild taro from Bangkok (from the same widespread Bangkok wild population sampled here) is interfertile with cultivated taro and could confer Taro Leaf Blight (TLB) resistance (Singh et al., 2012). This suggests a close genetic relationship between wild CIII taro and the cultivated taro parent (most likely CI in the Pacific). If CI and CIII taros are found to have morphological and genetic traits that set them apart, despite being interfertile, then taxonomic revision may be needed. *Caladium acre* (Brown, 1810–1830), an early name proposed for wild taro in northern Australia and discussed by Hay (1998), is of interest here as a possible precedent for renaming CIII wild taros.

### 4.2 Scenario 2: Long‐evolved, sister hybrid species

Wild CIII *C. esculenta* and *C. lihengiae* in our sample set (Figures 2 and 3) are sister hybrid species, both arising from crosses between *C. esculenta* as a paternal, nuclear genome donor and an unknown CIII species as a maternal chloroplast donor. These putative hybrid species differ in floral and vegetative morphology and habitat preferences, so the original hybridization event may have been long ago, giving time for separate evolution as species. Alternatively, there may have been multiple, early hybridization events (that is, reticulation) involving different ancestral populations of each parent species. In either case, the nuclear genomes of CIII wild taros should be distinct from the nuclear genomes of CI taros. This scenario is not supported by the morphological similarity of CI and CIII wild taros and the dissimilarity of the latter and *C. lihengiae* (assuming that most of the morphological differences that distinguish species are determined by nuclear genomes).

### 4.3 Scenario 3: Hybridization, introgression, and chloroplast capture followed by range extension (3.1) from west to east and south, or (3.2) from east to west and south

Commensal wild taros in Southeast Asia are often CI, entirely green with long stolons (e.g., Figures A2 and A3), and comparable to, or identified as, *C. esculenta* var. *aquatilis* (Ahmed et al., 2020). Wild CIII taros (e.g., Figures A5, A6, A7) share this overall morphology. Hybridization followed by introgression and chloroplast capture (Bock et al., 2014; Rieseberg & Wendel, 1993) may explain this overall similarity despite the clustering of CIII taro haplotypes with other wild *Colocasia* species. Previous estimates for divergence times between the CI, CII, and CIII lineages were all roughly 5–13 million years ago, during the Miocene (Ahmed et al., 2020). Although the estimates were very approximate, it is unlikely that the CI and CIII lineages are minor variants that evolved within a single species. As far as they are currently known, CI and CIII wild taros lack morphological traits that set them apart (contrary to *Scenario 1*), and this can be tested by giving closer attention to morphological diversity in wild taro populations. From the evidence of chloroplast diversity alone, the directionality of chloroplast capture and subsequent range extension cannot be determined, so we cannot be sure which haplotype, CI or CIII, is authentic or typical for *C. esculenta*. Two possible versions of this scenario are therefore proposed below.

#### 4.3.1 Range extension from west to east and south

Hybridization, introgression, and capture of a CIII chloroplast genome by a CI wild taro from the Assam–Bengal floodplain produced an invasive hybrid (“floodplain *C. esculenta*” × *Colocasia*? sp.) that spread east and south across the Sunda shelf and then further east to New Guinea and Australia. The CIII maternal chloroplast donor here may have been *C. lihengiae/mannii* or another wild CIII species in lower‐montane Southeast Asia. The nuclear genome of the hybrid may represent a subset of the nuclear genomic diversity in CI wild taros (“floodplain *C. esculenta*”), regardless of the long period required for CIII wild taros to spread from Southeast Asia to Australia and Papua New Guinea, and possible differentiation of the nuclear genome during that period.

#### 4.3.2 Range extension from east to west and south

Hybridization, introgression, and capture of a CI chloroplast genome by a CIII wild taro from lower‐montane Southeast Asia produced an invasive hybrid (“lower‐montane *C. esculenta*” × *Colocasia*? sp.) that spread westward across the Assam–Bengal floodplain and then southward into the Indian peninsula, where wild taro populations are also widespread. The CI maternal chloroplast donor in this scenario could have been an unknown CI species in Northeast India, Bangladesh, or another nearby region. The nuclear genome of the hybrid may represent a subset of nuclear genomic diversity in CIII wild taros (“lower‐montane *C. esculenta*”), and there could have been further divergence in the nuclear genome of CI wild taros if the proposed events were early enough. Chloroplast genome diversity in taro across the Indian peninsula remains entirely untested, but is assumed here to lie within the CI lineage.

Since many genetic and morphological parameters remain uncertain, we cannot favour any particular scenario at present or estimate the timings for the events proposed. There are also many possibilities for complex admixture of nuclear genomes that cannot be assessed here, especially if hybridization events have been common. Such events might have occurred long before human interactions with *Colocasia* spp., but human interactions with taro could also be very old, given the deep antiquity of modern humans and other hominins (*Homo* spp.) in tropical Asia and the ability of early modern humans to occupy tropical forest environments (Bacon et al., 2021; Roberts et al., 2016).

Previously, we found that CI, Type 1 taros, are widespread as tropical wetland cultivars and in commensal wild populations (Ahmed et al., 2020). The diversity of CI wild populations in Bangladesh (Figure 4) suggests that CI, Type 1 taros (here represented by *Ta‐imo*, a wetland cultivar in southern Japan, Figure 4 and Figure A3) might have originated in the extremely west Bengal delta. This raises a further question of how and when CI taros moved eastward or westward across the Brahmaputra/Burma boundary, a major terrestrial biogeographic barrier for plants and animals that may also have been a barrier for modern humans until the Last Glacial Period (beginning at Marine Isotope Stage 4, MIS 4, approx. 71,000 years ago), when human movement into Island Southeast Asia is thought to have been favoured by more open forest habitats (Boivin et al., 2013).

Hypotheses regarding the genetic and geographical origins of tropical wetland cultivars (CI) cannot be tested without geographical sampling of wild populations across the entire Bengal delta, upper catchments of the Ganga and Brahmaputra rivers, the Indian peninsula, and other regions around the Bay of Bengal. There are further huge gaps in sampling across Southeast Asia, East Asia, and the western Pacific. The likely route for dispersal of CIII wild taro through Indonesia to Australia and New Guinea (Ahmed et al., 2020) remains unexplored. This gap is unfortunate since Rumphius (2011) (1741–1750) (writing in the late 17th century, and first published in the 18th century) already distinguished two kinds of wild “water Kelady” (water taro, “Kelady Ayer”) by habitat and usage, namely: “*Vicorum*” [Latin for “village”], growing in mire (swampy, boggy ground) in and behind the villages of Ambon, and “seldom eaten” because it is “too sharp” (= CIII, a natural inland population?), and “*aquatile*”, growing on the sides of rivers on Ambon and Java, and often eaten by poor people or used as pig's fodder (= CI, an invasive, commensal wild population?). Throughout Southeast Asia, there are innumerable opportunities for localized, present‐day interbreeding between remnant natural populations of wild taro and commensal wild taro populations that have spread widely in open habitats created by humans (most notably in the vicinity of wetland rice production in drains, irrigation channels, and along streams and rivers). Ongoing interactions between wild taro populations and other *Colocasia* species are also likely, according to the presence and preferences of specialist insect pollinators (*Colocasiomyia* spp.) at different altitudes and among different potential hosts. Detecting past interactions among closely related *Colocasia* species will require more detailed population studies in areas with and without sympatry today.

## 5 CONCLUSIONS

The present study raises many biological, taxonomic, and historical questions. By using complete and intergenic chloroplast genome sequences (Figures 2 and 3), we have refined the model (hypothesis) of phylogenetic relationships among *Colocasia* species. The genealogical tree presented here is more robust than that reported by Ahmed et al. (2020), but is certain to change again in the future when more *Colocasia* species are added. Cultivated taros are now seen to belong to sister lineages (CI, CII) that form a distinct group with a deep evolutionary split, despite the morphological similarity of many wild CI taros to the wild CIII taro lineage. Since CIII chloroplast genomes are found in multiple wild species, those species may represent a major clade within *Colocasia*. In future surveys, CI and CII haplotypes might also be found in multiple species. It is also possible that inter‐specific hybridization has contributed to the evolution of new *Colocasia* species and reticulate evolution (Arnold, 2016) among closely related and widely distributed species such as *C. esculenta*, *C. lihengiae*/*mannii*, and others. Hybridization, chloroplast capture, and range extension could have happened long before the domestication and spread of taro as a crop, or more recently as part of crop history. Regardless of when and where such events first occurred, hybridization might have been accelerated by human translocation of fertile, diploid CI, and CII taros into new areas sympatric with each other and with other wild *Colocasia* species.

In the future, it may be necessary to recognize CI, CII, and CIII taros as distinct species, or *C. esculenta* as one species that includes diverse hybrids arising in nature and as a result of human activities. Formal taxonomic revision of *C. esculenta* will require a search for wild CII populations for comparison with wild CI and CIII populations, and a study of wild *Colocasia* species that may have been involved in the proposed process of hybridization, chloroplast capture, and range extension. Since taro is a globally cultivated crop with a wide‐ranging literature based on current taxonomy, care is needed to gain at least some acceptance for changes in nomenclature before they are formally proposed. Basic taxonomic issues concerning wild and cultivated taros were explored by Hay (1998), who recommended abandoning historical varietal names applied to cultivated forms of taro (var. *esculenta*, var. *antiquorum*). We use these names here for convenience, with the caveat that they do not represent the full range of morphological diversity in taro.

We recommend that *C. esculenta* (L.) Schott continue to be regarded as a single polymorphic species until more is known about nuclear genome diversity in wild taro populations and closely related wild *Colocasia* species. Complementary studies of chloroplast and nuclear genome diversity are needed to explore the evolutionary scenarios presented here, and wider sampling of populations is needed to confirm the “authentic” genomic components (Rieseberg & Wendel, 1993) of each species. A closer study of morphology and its development is also needed to better distinguish CI, CII, and CIII taros in the field and possible hybrid forms involving the different chloroplast genomes.

Experimental work to explore breeding barriers will help to resolve issues in the taxonomy of taro and *Colocasia* species and to identify species that can most easily or usefully contribute to crop breeding. Currently, there is no international collection of wild *Colocasia* species or international institution with a mandate for basic research on taro and its wild relatives. Breeding programmes for taro have been constrained for many reasons (Lebot & Ivančič, 2022), and the lack of basic research to address biological, taxonomic, and historical questions is also a significant constraint. These questions involve wild and cultivated species, evolutionary history, and deep human history, so holistic and integrative approaches (Hay, 2019) are needed across the natural, agricultural, and human sciences.

## AUTHOR CONTRIBUTIONS

**P. J. Matthews:** Conceptualization (lead); data curation (equal); formal analysis (equal); funding acquisition (lead); investigation (lead); methodology (equal); project administration (lead); resources (equal); visualization (lead); writing – original draft (lead); writing – review and editing (lead). **M. A. Hossain:** Investigation (supporting); resources (equal); writing – original draft (supporting). **D. Sookchaloem:** Investigation (supporting); resources (equal); writing – original draft (supporting). **V. D. Nguyen:** Investigation (supporting); resources (equal); writing – original draft (supporting). **S. Y. Wong:** Investigation (supporting); resources (equal); writing – original draft (supporting). **J. Joling:** Investigation (supporting); resources (equal); writing – original draft (supporting). **M. E. Schranz:** Writing – original draft (supporting); writing – review and editing (supporting). **F. T. Bakker:** Writing – original draft (supporting); writing – review and editing (supporting). **E. Tabuchi:** Data curation (equal); formal analysis (supporting); funding acquisition (supporting); methodology (supporting); project administration (supporting); visualization (supporting); writing – original draft (supporting). **I. Ahmed:** Conceptualization (supporting); data curation (equal); formal analysis (equal); methodology (equal); resources (supporting); writing – original draft (supporting); writing – review and editing (supporting). **A. Hay:** Writing – original draft (supporting); writing – review and editing (supporting).

## FUNDING INFORMATION

P.J. Matthews was supported by JSPS Kakenhi Grants 17H04614 (P. J. Matthews, National Museum of Ethnology, Osaka), 17H01682 (K. Watanabe, Tsukuba University, Tsukuba), and 20K20504 (R. Ono, National Museum of Ethnology). V.D. Nguyen was supported by the Vietnam National Foundation for Science and Technology Development (NAFOSTED) Grant 106.03‐2019.322. S. Y. Wong (UNIMAS) was supported by Shell Chair Grant (Universiti Malaysia Sarawak) I01/SHC/1962/2020. I. Ahmed was supported by a JSPS Short‐term Invitational Fellowship (2022) and a Brain Pool Fellowship by the National Research Foundation of Korea (2023‐25; Grant RS‐2023‐00223245).

## CONFLICT OF INTEREST STATEMENT

The authors have no conflicts of interest to declare.

## PERMISSION TO REPRODUCE MATERIALS FROM OTHER SOURCES

No other sources used.

## Figures and Tables

**FIGURE A13:**
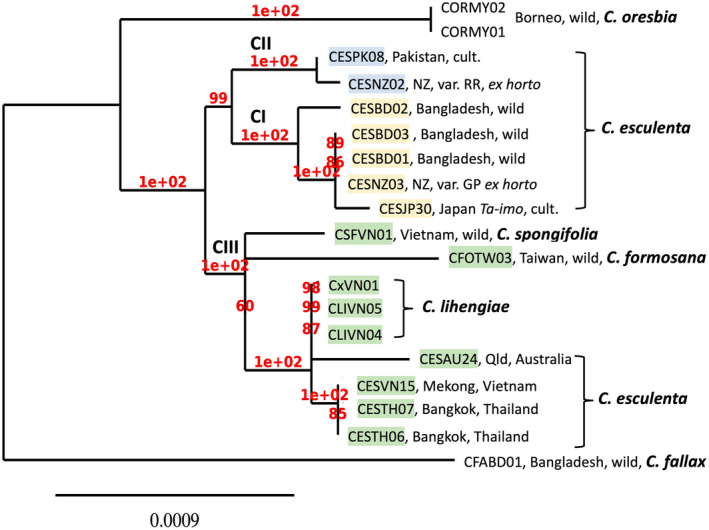
Original IQ‐Tree output with bootstrap values for ML tree with *C. fallax* and five other *Colocasia* species, based on all protein‐coding gene sequences (CDS), excluding those of IRa (one of two copies of the large inverted repeat). See final tree in Figure A12.

## Data Availability

All sample collection data are provided in the Appendix A (Table A1). All aligned sequence data are available under the title “Complete, coding and intergenic sequences from the chloroplast genomes of six *Colocasia* species, in three alignments” (Dryad, doi:10.5061/dryad.3n5tb2rqk). Complete, annotated chloroplast genome sequences for all 17 newly sequenced samples are available at Genbank (accession numbers in Table A1).

## 1

This appendix has four sections: 5.1. Sample illustrations (Figures A1, A2, A3, A4, A5, A6, A7, A8, A9, A10, A11) showing sampled plants, populations, and habitats. 5.2. A sample list (Table A1) with sequence‐related summary data and sample collection details. 5.3. Method details with further ML tree diagrams (Figures A12, A13, A14, A15) and tree comparison statistics (Table A2). 5.4. Additional discussion of methodological constraints, substitution models, and taxonomic sampling.

### 1.2 Sample list

The following sample list (Table A1) includes sample ID labels, Genbank sequence accession numbers, sequence size, label history, and collection notes for each sample. Sample descriptions are based primarily on written field notes (Matthews and other collectors). Photographic records for each sample (not including that from Pakistan) are provided in Figures A1, A2, A3, A4, A5, A6, A7, A8, A9, A10, A11. GPS latitude and longitude data were converted to a full decimal format for submission to Genbank using Google Earth (GE). At the same time, elevation data provided by the GPS were cross‐checked with GE satellite images, and in some cases, the elevation indicated by GE appeared more reliable based on direct experience of each site in the field; in such cases, the GE figure is indicated.

**TABLE A1:** Sample list.

Line no.	Sample label, Genbank no., assembled genome size, & haplogroup	Name	AG label	NME museum label	Collection notes	Photos
1	**CESNZ03** JN105689 162,424 bp, CI	*C. esculenta* var. GP (Matthews, 1985, 2014)	na	na	Campus garden (*ex horto*), University of Auckland. Coll. I. Ahmed, 25th June 2008 (IA004, Massey University herbarium MU004). Introduced to New Zealand at unknown date; commonly wild around settlements (Matthews, 1985, 2014). Analysed by Ahmed et al. (2012, 2013, 2020). Vic. 36.8517 S, 174.7723 E, elevation ca. 24 m (GE)	Figure A1a,b
2	**CESBD01** PP811809 162,424 bp, CI	*C. esculenta*	WP396	WP396, list BD02	Wild, commensal, on upper bank of Old Brahmaputra (river), Mymensingh, Bangladesh. Coll. PJM & M. A. Hossain, 10th Feb. 2019. Isolated purple plant near scatter of all‐green wild taros, and fenced garden at top of the bank (likely an escaped cultivar); blade upper surface dark green, lower surface pale green, with some purpling of ribs and tertiary veins; petiole dark purple to purple‐green, with white base, white basal ring; corm large, starchy (estimated 2 years growth), with white skin, white cortex and core, thick white roots, and stolon. GPS 24.7230 N, 90.4441 E, elevation (GE) 12 m	Figure A1c,d
3	**CESBD02** PP817734 162,463 bp, CI	*C. esculenta*	WP475	WP475, list BD20	Wild, commensal, Sylhet, Bangladesh. Coll. PJM & M. A. Hossain, 13th Feb. 2019. All green blade and petiole; blade angular with deep sinus and undulating margin; petiole base and basal ring pale green; corm with stolons and white roots, skin, cortex, and core parenchyma; on damp ground between road and open, grazed field; forest remnant nearby has wild *Alocasia* spp., *Steudnera* sp. (Colocasiodeae). GPS 24.5136 N, 91.9942 E, elevation (GE) 40 m	Figure A2
4	**CESBD03** PP817735 162,386 bp, CI	*C. esculenta*	WP501	WP501, list BD26	Wild, commensal, form A, “*shak kasu/kochu*” (leaf taro), Sylhet, Bangladesh. Coll. PJM & M. A. Hossain, 14th Feb. 2019. Single clump of all‐green form (in patch with black “form B” “*kalo kasu/kochu*” = “dark taro”, which was predominant at this site, also with stolon). Leaf all green; blade angular with deep sinus, tertiary veins on underside not raised (surface smooth); petiole base pale green, corm undeveloped (young plant), with stolon beginning to emerge. Taro plants generally scattered on uneven ground under trees and on bund of a small fish pond. Local informants: both colour forms can be eaten, but they prefer “white” (i.e. pale green) form; neither form produces corms in wild (culinary use of wild *shak* is common knowledge in this region). GPS 24.5617 N, 90.7430 E, elevation (GE) 8 m	Figure A3a–c
5	**CESJP30** PP817736 162,376 bp, CI	*C. esculenta*	CV_CP	cv *Ta‐imo*	Cultivar ex pond field, vicinity Kamkatetsu‐Keraji, Kikai island, Amami, Japan. Coll. PJM & E. Takei, 14th March 2017. Leaf sample from plant in FSL laboratory, 7th Jan. 2020; identified from literature as cv. *Ta‐imo* “red”; plant w. pink basal ring, pink buds, and pink skin; dense texture and sweet flavour when lab‐grown corm cooked (2022). Vic. 28.2820 N, 129.9481 E, elevation 7 m (GE)	Figure A3d–f
6	**CESNZ02** JN105690 162,546 bp, CII	*C. esculenta* var. RR (Matthews, 1985, 2014)	na	na	Campus garden (*ex horto*), University of Auckland, Auckland. Coll. I. Ahmed, 25th June 2008 (IA003, Massey University herbarium MU003); var. RR. (a common cultivar, also planted and spreading on stream banks; Matthews, 1985, 2014). Analysed by Ahmed et al. (2012, 2013, 2020). Vic. 36.8517 S, 174.7723 E, elevation ca. 24 m (GE)	Figure A4
7	**CESPK08** PP817737 162,478 bp, CII	*C. esculenta*	TLP_CP	na	Cultivated, ex market, Islamabad, Pakistan. Coll. I. Ahmed, 2020. Small side corm with sprout used for extraction. Nominal locality, Central PO 33.7183 N, 73.0845 E, elevation 561 m (GE)	No photo
8	**CESVN15** PP817740 162,641 bp, CIII	*C. esculenta* c.f. var. *aquatilis*, (Matthews, 1991, 2014; Matthews et al., 1992)	WP734	WP734, list VN09	Wild, commensal, in tall grass on canal bank; on settled, rural delta island between main Mekong river and Song Hau, Mekong, Vietnam. Coll. PJM & Nguyen V. D., 21st Sept. 2017. Sample site is part of a widespread wild population in the area (plants locally known as *mon ngua*, “itchy taro”). Plants at sample site displayed fully mature orange fruit; cocoons of the pollinator fly (*Colocasiomyia*) were also present on fruiting heads. Resident of adjacent house described the plant as having no use; other residents nearby (at WP737, Matthews field notes) described a long process (peeling, salting, soaking) previously used to make petioles edible; the petioles were previously sold in market but other vegetables are now preferred. GPS 10.3545 N, 105.6688 E, elevation 5 m	Figure A5
9	**CESTH06** PP817738 162,644 bp, CIII	*C. esculenta* c.f. var. *aquatilis*, (Matthews, 1991, 2014; Matthews et al. 1992)	WP509	WP509	Wild, Khlong Prapa, Bangkok, Thailand. Coll. PJM & D. Sookchaloem, 18th Feb. 2019; spreading clump in ditch with *Typha*, in vacant land under raised motorway, approx. 500 m from old side bend of Chao Praya; fruiting; leaf all green with white petiole base, white basal ring; roots and corm skin, cortex and core white; stolons long. GPS 13.9821 N, 100.5702 E, elevation (GE) 2 m	Figure A6a–c
10	**CESTH07** PP817739 162,644 bp, CIII	*C. esculenta* c.f. var. *aquatilis*, (Matthews, 1991, 2014; Matthews et al. 1992)	WP516	WP516	Wild, Ko Kret Island, Chao Praya, Bangkok, Thailand. Coll. PJM & D. Sookchaloem 18th Feb. 2019; “*bai bon*”, not eaten; flowering, leaves all green; spreading with stolons in bog behind waterfront houses. GPS 13.9055 N, 100.4901 E, elevation (GE) 5 m	Figure A6d,e
11	**CESAU24** PP475493 162,584 bp, CIII	*C. esculenta* var. *aquatilis* (Matthews, 1991, 2014)	L1	Extract tube L1.3 (= field sub‐location number)	Wild, Hopevale, Queensland, Australia. Coll. PJM & K. Thiele, 26th Sept., 1987; leaves all green; plants spreading with stolons in seasonal stream and perennial wet seepage area. See site description in Hunt et al. (2013). Vic. 15.296 S 145.112 E, elevation 45 m. (Hopevale settlement location; site is about 5 m lower close to stream)	Figure A7
12	**CLIVN04** PP817743 162,557 bp, CIII	*C. lihengiae* (Long & Liu, 2001)	93_CP	WP 933B, list VN93	Wild, commensal; Xuan Son Guest House, Phu Tho prov., Vietnam. Coll. PJM & Nguyen V. D., 4th Oct. 2017. Plants scattered at edge of clearing and forest, in gully behind house, together with wild *C. esculenta*; *C. lihengiae* described by resident as edible (recipe recorded). GPS GPS 21.1261 N, 104.9560 E elevation 415 m	Figure A8a,b
13	**CLIVN05** PP817744 162,640 bp, CIII	*C. lihengiae* (Long & Liu, 2001)	107_CP	WP 944A, list VN107	Wild, Tukuk Commune, Dang Son district, Vietnam. Coll. PJM & Nguyen V. D., 4th Oct. 2017. With Pu petiole, In small open gully next to road. GPS 21.2318 N, 104.9490 E, elevation (GE) 105 m	Figure A8c–e
14	**CxVN01** PP817748 162,641 bp, CIII	*C. lihengiae* × *C. sp. C. menglaensis* = suspected male parent	114_CP	WP964, list VN114	Wild, roadside, Yen Bai/Phu Tho border. Coll. PJM & Nguyen V. D., 5th Oct., 2017. Noted in field as possible hybrid, *C. lihengiae* × ?*C. menglaensis*. Flowering not seen. GPS 21.3659 N, 104.8562 E, elevation 374 m. (Approx. 29 km distant from CLIVN04 collection site and 16 km from CLIVN05, cf. genetic distances in Figure 4)	Figure A8f–i
15	**CSFVN01** PP817747 162,388 bp, CIII	*C. spongifolia* (Matthews et al., 2022)	CSP_CP	WP251	Sample from seedling grown ex situ; seed ex wild plant, Bach Ma National Park, Vietnam. Coll. PJM and Nguyen V. D., 22nd Sept. 2018. Leaf all green; petiole green to base; petiole sheath broad, no stolon (buds only); abundant fruiting; on wet ground at base of slope between road and forest, growing on granite rubble and humus; associated flora incl. *Begonia*, *Elatostema*, tree fern, wild banana, *Alocasia odora* (epiphytic on wall of ravine with waterfall). 16.2043 N, 107.8579 E, elevation 936 m	Figure A9
16	**CFOTW03** PP817742 162,158 bp, CIII	*C. formosana* (Hayata, 1919; Hsu et al., 2000)	CFO_CP	WP108	Seedling grown *ex situ*; seed ex wild plant, Wutai district, Pingtung County, Taiwan. Coll. PJM and K.‐C. Tsai, 1st Sept. 2014. Abundant plants around waterfall; flowering, bees and pollinating flies present (also collected). In this area, the plant is known as famine food requiring special care to cook. GPS 22.7351 N, 120.7396 E, elevation (GE) 1030 m	Figure A10
17	**CORMY01** PP817745 161,973 bp, outgroup	*C. oresbia* (Hay, 1996)	JJ04	JJ04	Wild, Jalan Tambunan‐Kota Kinabalu, Tambunan, Sabah, Malaysia. Coll. J. Joling 22nd Dec. 2020. GPS 5.8382 N, 116.3318 E, elevation ca. 1547 m	Figure 2
18	**CORMY02** PP817746 161,947 bp, outgroup	*C. oresbia* (Hay, 1996)	JJ07	JJ07	Wild, Mahua waterfall near entrance, Tambunan, Sabah, Malaysia, Coll. J. Joling 22nd Dec. 2020. GPS 5.7975 N, 116.4036 E, elevation ca. 1100 m	Figure 2
19	**CFABD01** PP817741 161,252 bp, outgroup	*C. fallax* Schott (Ara, 2000; Deva & Naithani, 1985)	WP466A	WP466A, list BD13	Wild along stream bank below waterfall, Madhabkunda, Bangladesh. Coll. PJM & M. A. Hossain, 13th Feb. 2019. Main population abundant on steep slopes/rock surfaces, around waterfall; other flora incl. *Calamus*, *Elatostema*, wild banana, and *Colocasia* sp. (unknown); blade rounded with shallow sinus, not wettable (waxy); upper surface bluish‐green with pale blush alongside primary anterior vein; raised tertiary veins on underside; petioles green to purplish green to purple; stolons abundant, thin (3–4 mm diam.), wiry (strong); flowering not seen. GPS vic. 24.6380 N, 92.2243 E, elevation (GE) 70 m	Figure A11

*Note*: Genbank sample labels follow the series established by Ahmed et al. (2020) and employ the same species‐country‐number format, with a three‐letter code for each species (e.g., CES for *C. esculenta*), a two‐letter international country code, and an individual number. Label history: Alphagenomics Ltd (AG), National Museum of Ethnology (NME). WP, Garmin GPS waypoint site number. Latitude, longitude and elevation are based on the GPS and World Geodetic System WGS84 grid, unless noted as a vicinity estimate. Google Earth (GE) (2024) was used to visually check GPS elevations, and in some cases the GE estimate was preferred.

### A.3. Method details

The result of a preliminary analysis with *C. fallax* (Figure A12), original tree diagrams, log data from IQ‐Tree runs, and our derived calculations are all presented here. The run program used was *IQ‐TREE multicore version 1.6.12 for Linux 64‐bit, built Aug 15* ^*th*^ *, 2019*.

To prepare Figures 3 and 4, Figure A12, branches on the original trees produced by IQ‐Tree were rotated and swapped for a consistent layout. Figures A13, A14, A15 show the original trees with all bootstrap values and no manipulation of branches.

For each tree below (Figures A13, A14, A15), complete original sequence data were aligned, the Inverted Repeat‐a (IRa) region was deleted, the specific regions to be analysed were extracted and concatenated, and all gaps were removed. Original IQ‐Tree results (output trees with bootstrap values) are shown below and are used to calculate average bootstrap (BS) values across all nodes in each tree. To compare the data support and robustness of each ML analysis, with respect to sequence partition and model selection, calculations based on run log data are summarized in Table A2. Run log data are shown in full with each tree. Results obtained with the Jukes‐Cantor (JC) model (implemented in IQ‐Tree with ML optimized) are also shown in Table A2.

The average number of parsimony informative (PI) sites per 100 bp of aligned sequence, and average bootstrap (BS) percentages across all nodes were highest in our analysis of four closely related (in‐group) taxa using only the intergenic (non‐coding) sequence data (Table A2 and Figure A15). Overall, the analyses are similarly robust, but resolution of subclades within closely related taxa of the in‐group (CI–CIII, our main focus) was improved by removing outgroup taxa (*C. fallax*, *C. oresbia*) and the most conserved component of the chloroplast genome (CDS).

In Figures A13 and A14, *C. formosana* is shown as a near outgroup for CIII *C. esculenta* and *C. lihengiae*, with low bootstrap values (60% and 52%, respectively), while in Figure A15, *C. spongifolia* is shown as the near outgroup, with a low bootstrap value of 58%. The conservative CDS analysis (Figure A13) may be the most reliable indication of phylogeny in this case, but more study is needed. Since the entire haplogroup with C*. spongifolia* and C*. formosana* includes an apparent polytomy that cannot be resolved into distinct lineages, the haplogroup is identified here as just one CIII lineage.

**TABLE A2:** Summary of calculations based in IQ‐Tree run log data.

Genome component	No. species	Aligned bp	No. PI sites	PI/100 bp	Av. BS%	Figures	Model
CDS	6	68,985	206	0.300	93.94	A12, A13	TVM + F + I
					93.56	–	JC, ML opt.
Complete	5	134,382	696	0.518	93.90	3, A14	K3Pu + F + I
					94.56	–	JC, ML opt.
Intergenic	4	46,269	249	0.538	94.80	4, A15	K3Pu + F + I
					95.36	–	JC, ML opt.

*Note*: All analyses with IRa and gaps removed; CDS = protein coding sequence, “Complete” = all data after removal of IRa and gaps; “Intergenic” = all non‐coding exons (no introns included). Average bootstrap (BS) values are shown for each substitution model selected by ModelFinder, and for the Jukes‐Cantor model (selected manually). Models used were: Kimura 3‐parameter (K3P) model with variable base frequencies, equal transition rates, two transversion rates; Transversion model (TVM) with variable base frequencies, variable transversion rates, transition rates equal, and Jukes‐Cantor (JC) with equal base frequencies, all substitutions equally likely.

For each IQ‐Tree analysis in which ModelFinder selected the best fit model, the run log data and calculations are given below, after each original output figure (Figures A13, A14, A15).

*Details and calculations for Figure* *A13* *(and A12, which is based on A13 after branch swapping)*. Run date: 2023‐04‐13. Run log data: Alignment has 19 sequences with 68,985 columns (bp), 199 distinct patterns, 206 parsimony‐informative sites, 264 singleton sites, and 68,515 constant sites. Best‐fit model: TVM + F + I chosen according to BIC. The run generated 1000 samples for ultrafast bootstrapping. All distinct (visible) nodes on the tree have bootstrap support of 100%, except for one with 99%. Calculations: Average PI = no. parsimony‐informative sites/bp ×100 = 206/68,985 = 0.300 sites/100 bp. Average BS = sum bootstrap percentages/no. nodes = 1503/16 = 93.94 % per node.

*Details and calculations for Figure* A14. Run date: 2022‐07‐11. Run log data: Alignment has 18 sequences with 134,382 columns (bp), 279 distinct patterns, 696 parsimony‐informative sites, 392 singleton sites, and 133,294 constant sites. The best‐fit model: K3Pu + F + I, was chosen according to BIC. The run generated 1000 samples for ultrafast bootstrap. All distinct (visible) nodes on the tree have bootstrap values of 100%. Calculations: Average PI = no. parsimony‐informative sites/bp ×100 = (696/134,382)100 = 0.518 sites/100 bp. Average BS = sum bootstrap percentages/no. nodes = 1503/16 = 93.9% per node.

*Details and calculations for Figure* *A15*: Run date: 2022‐07‐11. Run log data: Alignment has 16 sequences with 46,269 columns, 173 distinct patterns, 249 parsimony informative, 250 singleton sites, and 45,770 constant sites. The best‐fit model: K3Pu + F + I, was chosen according to BIC. The run generated 1000 samples for ultrafast bootstrapping. Most distinct (visible) nodes on the tree have bootstrap values of 100%, and none have values less than 83%. Calculations: Average PI = no. parsimony‐informative sites/bp ×100 = (249/46,269)100 = 0.538 sites/100 bp. Average BS = sum bootstrap percentages/no. nodes = 1327/14 = 94.8% per node.

### A.4. Methodological constraints, substitution models, and taxonomic sampling

Following precedents in other taxonomic studies (for example, Androsiuk et al., 2020; Duvall et al., 2016), various methods of analysis and substitution model selection were tried with the complete, unpartitioned chloroplast sequences. Maximum Likelihood (ML) analyses with different models in the General Time Reversible (GTR) family of nested models all gave similar results. Using unpartitioned data is known to be unrealistic because different regions mutate in different ways at different rates (Kelchner, 2000, 2008). Nevertheless, the long sequence (134,382 bp) (complete genome with one large inverted repeat removed) had many parsimony‐informative sites (Table A2) and gave a robust topology for the present taxonomic sample set (Figure 3 and Figure A14). For the CDS analysis (Figures A12 and A13) and intergenic sequence analysis (Figure 4 and Figure A15), the data were partitioned prior to model selection and analysis.

After using ModelFinder to identify the best‐fit substitution models to analyse our sample sets and data partitions, we also analysed each sample set using the Jukes‐Cantor (JC) model with ML optimization in IQ‐Tree (Table A2) in order to learn if the JC assumption of equal substitution rates at all positions affected the topology. Only small differences in the topology and branch lengths of each tree were found (diagrams not shown). Although the JC model gave better bootstrap support values for intergenic sequences, this substitution model was not selected by ModelFinder. In a series of experiments with simulated data sets, Abadi et al. (2019) found that the JC model generates topologies that are only slightly less often correct than those generated with complex models, and that the most complex nucleotide substitution model, GTR + I + G, consistently leads to inferences of tree topology that are very similar to those obtained with other models selected as optimal by jModelTest across a range of model selection criteria (BIC and others). They suggest that for many data sets, optimal model selection is not necessary and that the GTR + I + G model is adequate. We did not attempt to use models specific to putative functional domains of intergenic sequences (e.g., stem‐loop structures involved in gene regulation), as the main limiting factor for our study was the non‐availability of *Colocasia* species for testing.

Although the topologies appear robust for the present data set, topologies, and the biological significance of the results can be improved in the future by wider geographic sampling for each species and by more comprehensive taxonomic sampling of *Colocasia* species. There is no international collection of taro that can provide samples of close wild relatives. Our sample set represents approx. one quarter of known *Colocasia* species (Matthews et al., 2022), and it is likely that not all *Colocasia* species have been discovered, given the lack of botanical exploration for this genus in large areas of Southeast Asia.
